# Thickness Effect on Some Physical Properties of RF Sputtered ZnTe Thin Films for Potential Photovoltaic Applications

**DOI:** 10.3390/nano11092286

**Published:** 2021-09-02

**Authors:** Dumitru Manica, Vlad-Andrei Antohe, Antoniu Moldovan, Rovena Pascu, Sorina Iftimie, Lucian Ion, Mirela Petruta Suchea, Ştefan Antohe

**Affiliations:** 1 Faculty of Physics, University of Bucharest, 077125 Magurele, Romania; dumitru.manica@inflpr.ro (D.M.); vlad.antohe@fizica.unibuc.ro (V.-A.A.); sorina.iftimie@fizica.unibuc.ro (S.I.); lucian@solid.fizica.unibuc.ro (L.I.); 2 National Institute for Laser, Plasma and Radiation Physics, 077125 Magurele, Romania; antoniu.moldovan@inflpr.ro (A.M.); rovena.pascu@inflpr.ro (R.P.); 3 Institute of Condensed Matter and Nanosciences, “Université Catholique de Louvain”, B-1348 Louvain-la-Neuve, Belgium; 4 Center of Materials Technology and Photonics, School of Engineering, Hellenic Mediterranean University, 71410 Heraklion, Greece; 5 National Institute for Research and Development in Microtechnologies, 023573 Bucharest, Romania; 6 Academy of Romanian Scientists, 050044 Bucharest, Romania

**Keywords:** ZnTe, thin films, rf-magneton sputtering, AFM, surface metrology, thickness effect

## Abstract

Zinc telluride thin films with different thicknesses were grown onto glass substrates by the rf magnetron sputtering technique, using time as a variable growth parameter. All other deposition process parameters were kept constant. The deposited thin films with thickness from 75 to 460 nm were characterized using X-ray diffraction, electron microscopy, atomic force microscopy, ellipsometry, and UV-Vis spectroscopy, to evaluate their structures, surface morphology, topology, and optical properties. It was found out that the deposition time increase leads to a larger growth rate. This determines significant changes on the ZnTe thin film structures and their surface morphology. Characteristic surface metrology parameter values varied, and the surface texture evolved with the thickness increase. Optical bandgap energy values slightly decreased as the thickness increased, while the mean grains radius remained almost constant at ~9 nm, and the surface to volume ratio of the films decreased by two orders of magnitude. This study is the first (to our knowledge) that thoroughly considered the correlation of film thickness with ZnTe structuring and surface morphology characteristic parameters. It adds value to the existing knowledge regarding ZnTe thin film fabrication, for various applications in electronic and optoelectronic devices, including photovoltaics.

## 1. Introduction

There is a need for new nanostructured materials with enhanced properties, due to the development of new systems and materials that use nanotechnologies. Due to characteristics obtained at the time of the deposition process such as: low resistivity, high transparency in the visible spectrum, etc. [1,2,3,4], thin layers of zinc telluride (ZnTe) are used in various modern technologies, which are implemented in various micro- and nano-structured devices, such as light emitting diodes, solar cells, photodetectors, etc. [1,2,3,4,5,6,7,8,9,10,11,12,13,14,15,16,17,18]. Although it is a popular material, there are few publications and studies regarding its material engineering. ZnTe is a sensitive material in the green spectral region, with a bandgap of 2.26 eV and a low electronic affinity of 3.53 eV; it can be used as a p-type buffer material in hetero-junction solar cells based on CdTe [10,11,12,13,18]. It can be used as back contact material to CdTe-based solar cells [14] in a multilayer device. It is a precious material from an ecological point of view; it can be used as a replacement for the CdS window layer [15]; the “condition” is that the conductivity is n-type. For the deposition of thin layers [16,17,18,19,20], more physical and chemical growth paths are known, such as molecular beam epitaxy (MBE) [21], chemical bath deposition (CBD), electron-beam evaporation, thermal evaporation, magnetron sputtering [12], and electrodeposition [19,20,21,22]. The ZnTe thin layer properties depend directly on the deposition method, deposition conditions, and the growth direction imposed by the substrate when the substrate is crystalline [5,6,7,8,23]. Some studies have established the ideal deposition conditions for various deposition methods, to obtain ZnTe layers with the necessary physical properties, a well-defined morphology, and crystal structure, but there is no systematic reproducible technology available for high performance ZnTe coating fabrications [2,3,4,5,6,7,8,9,24,25]. In this context, studies regarding correlation for growth conditions/parameters with ZnTe structuring and morphology, in correlation with their physical properties, are still very important.

ZnTe thin films presented in this scientific report were prepared by rf magnetron sputtering, varying the deposition time that was related to the deposition rate, and finally determining the film thickness. Depending on the selected conditions, we sought to obtain an intermediate layer for the formation of junctions and the facilitation of electric charge transfer, which can be used directly in multilayer solar cells. In order to understand the ZnTe film structuring during growth, the investigation was centered on structure and surface morphology evolution, and the surface characteristic parameters correlation with material optical properties, and how it changed depending on the deposition parameters. For this scope, the following characterization methods were used: scanning electron microscopy (SEM), X-ray diffraction (XRD), atomic force microscopy (AFM), ellipsometry (spectroelipsometer-SE), and UV-Vis absorption spectroscopy. SEM was used for films thickness evaluation in the cross section while XRD was used to verify material crystallinity. AFM in non-contact mode was used to characterize the surface morphology and topology. A WVASE spectroelipsometer with variable incidence angles (60°, 65°, and 70°) was used for optical characterization. Optical models were generated by WVASE32 software; the parameters n (refractive index), k (extinction coefficient), and roughness were measured by mounting the ellipsometer parameters Psi (Ψ) and delta (Δ).

## 2. Experimental Procedures

### 2.1. Fabrication Technique

Magnetron sputtering is an easy-to-use deposition method. Due to the simplicity and easy handling of the installation, it allows good control of the deposition parameters. These parameters are enclosure pressure, substrate target distance, substrate temperature, applied power in the deposition process, deposition time, etc. ZnTe thin films were grown onto BK7 optical glass substrates (Heinz Herenz, Hamburg, DE6, Germany). Prior to any deposition process, the substrates were cleaned in acetone (Chim Reactiv S.R.L., Bucharest, BUC, Romania) and isopropyl alcohol (Chim Reactiv S.R.L., Bucharest, BUC, Romania) for 15 min, for each procedure, and then were rinsed in deionized water dried under nitrogen flow.

For all fabricated samples, the substrate-target distance, working pressure, substrate temperature, and applied power were maintained constant at 10 cm, 0.86 Pa, 250 °C, and 100 W, respectively, and the deposition time varied at 5, 10, 15, and 20 min. For ease of discussion, in the whole manuscript, the samples were denoted ZnTe1 (5 min), ZnTe2 (10 min), ZnTe3 (15 min), and ZnTe4 (20 min). 

### 2.2. Characterization Methods

In order to characterize the obtained thin layers, various characterization techniques were involved. The characterization techniques used were SEM, AFM, UV-Vis, SE, and XRD.

#### 2.2.1. Morphological and Structural Characterization

SEM was used to study the film cross sections and to estimate their thickness. While AFM was used for detailed characterization of film surfaces.

#### 2.2.2. SEM Characterization

The SEM cross-section micrographs were obtained in high vacuum by using a Tescan Vega XMU-II electron microscope (Brno-Kohoutovice, B, Czech Republic) operating at 30 kV with a detector for secondary electrons.

#### 2.2.3. AFM Characterization

The morphology of the surface was analyzed by AFM topography measurements using a XE100 AFM, from Park Systems (Suwon, Republic of Korea). The measurements were carried out in a noncontact mode, using silicon cantilevers (PPP-NCHR, Nanosensors, Neuchatel, Switzerland). The surface was scanned into various areas of the films with sizes of 5 μm × 5 μm and 2 μm × 2 μm. The surface roughness characteristic parameters were estimated using the AFM software while the surface topology specific parameters and texturing were evaluated by using Gwyddion version 2.49 (2017) open access specialized SPM software [26].

#### 2.2.4. X-ray Diffraction

The structural features of fabricated ZnTe films were investigated by XRD, using a Bruker D8 Discover diffractometer from Brucker (Bruker Nano GmbH Am Studio 2D, 12489 Berlin, Germany) using CuKα = 1.54 Å radiation in Bragg–Brentano theta–theta geometry. The scattered X-ray photons from samples were recorded in the 2θ range of 20°–70° with a scanning rate of 0.04°/s at room temperature.

#### 2.2.5. Optical Characterization

##### Spectrophotometry (UV-Vis)

The optical properties of thin films depend on the structure, composition, and physical and chemical properties of the material. Using UV-Vis optical spectroscopy, information about the structure of energy levels and bands and photoconduction mechanisms can be obtained. UV-Vis optical spectroscopy transmission measurements were performed in the wavelength ranges of 300–1500 nm, at room temperature, using a Lambda 750 spectrophotometer from Perkin Elmer (Norwalk, CT, USA).

##### Ellipsometry

One of the widely used characterization methods was ellipsometry. For the optical characterization of ZnTe samples, a Spectro-ellipsometer WVASE, (Lincoln, NE, USA) was used, with variable angels of incidence (60°, 65°, and 70°), for optical characterization, having high accuracy and precision with a wide spectral range of 250–1700 nm. Optical models were generated by WVASE32 software; n, k parameters, and roughness were measured by fitting the Psi (Ψ) and delta (Δ) parameters.

## 3. Results and Discussions

### 3.1. SEM Microscopy Analysis—Film Thickness Evaluation

**Figure 1:**
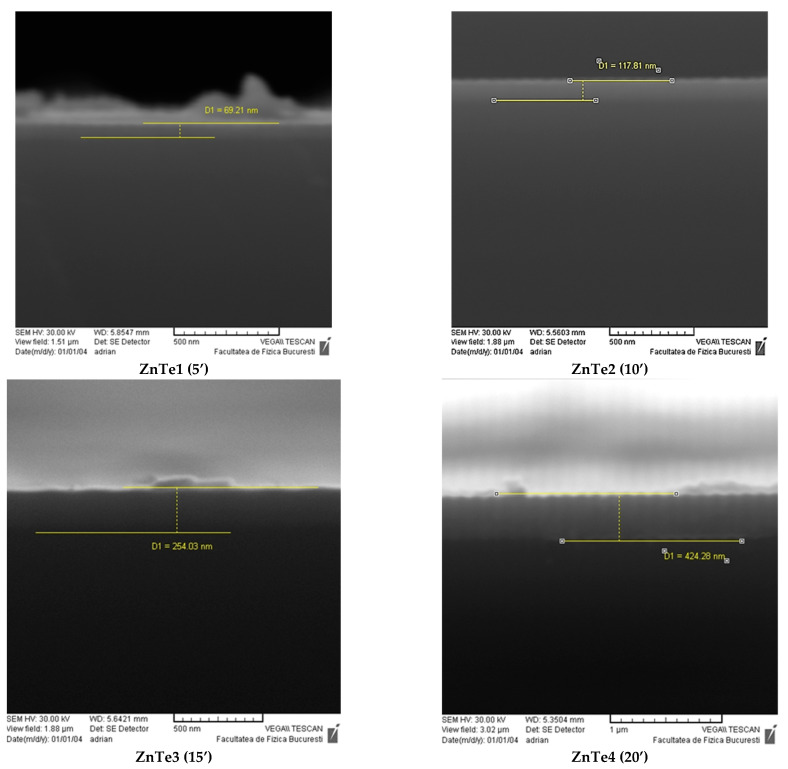
Examples of SEM images of cross sections of the fabricated ZnTe thin films (a local thickness estimation for each kind of film—the difference from the average values is due to local variations and it is in the errors limits).

All samples were characterized by SEM microscopy as described above. The SEM analysis shows the effect of the deposition parameters on the material structuring. For example, Figure 1 presents representative images of layers grown under similar conditions, except for the variation of the deposition time. One can see from the SEM characterization how the structuring evolves when the deposition time changes. The ZnTe layers are compact with a relatively constant thickness. Some large asperities can be observed on the thinner film surface. As the film grows, the surface becomes cleaner and the layers seem to become more compact. Using the SEM analyses options, the local thickness of the ZnTe layers deposited by rf magnetron sputtering vas estimated. To evaluate film thicknesses, a mean value was calculated using local values, measured as shown in the examples presented in Figure 1, in different locations along the substrate lengths. Mean thickness values (calculated as the arithmetic average of various local thickness measured along the cross sections of each film) were estimated to be ZnTe1 (5 min) 75 nm, ZnTe2 (10 min) 154 nm, ZnTe3 (15 min) 251 nm, and ZnTe4 (20 min) 461 nm, respectively, with an error bar of ± 10% for each value.

It was noticed that, with the increase of the deposition time, the thickness of the thin layers also changed. It can be observed that the growth rate slightly increased from 15 nm/minute for 5 min growth time; 15.4 nm/min for 10 min; 16.3 nm/min 15 min and becomes 23 nm/min for the thickest film. The increase of the growth rate in time can be attributed to the evolution of the growth mechanism of the films onto the substrate. In the early stage of nucleation, an island growth mechanism is present. This growth can be noticed when the adherence between the atom to atom is greater than the bonding between the substrate and the adatoms; it was observed on the thinnest films where the film was rough, island-like structured, and the thickness had larger variations across the substrate. At longer deposition times, the adatoms begin to accumulate; migration took place and the ZnTe layers with enhanced crystallinity were formed, as can be seen in Figure 1, and confirmed further by XRD analysis (see Section 3.2. Structural Characterization, X-Ray Diffraction).

### 3.2. Structural Characterization, X-ray Diffraction

**Figure 2:**
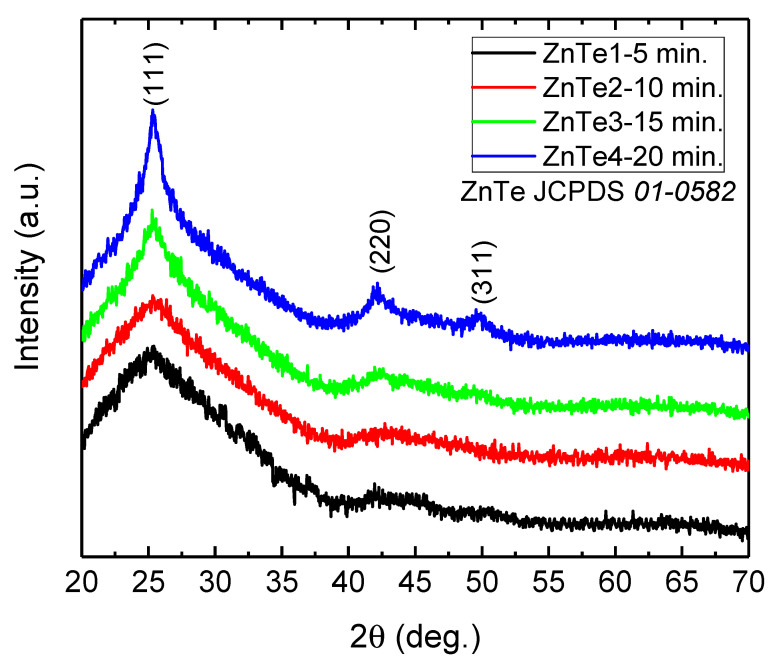
X-ray diffractograms of ZnTe films with different thicknesses.

X-ray diffraction patterns were recorded and diffractograms for the samples were obtained at different deposition times: 5, 10, 15, and 20 min, labeled as ZnTe-5 min, ZnTe-10 min, ZnTe-15 min, and ZnTe-20 min, are presented in Figure 2. Diffraction features located at 25.24°, 42.34°, and 49.59° can be assigned unambiguously to (111), (220), and (311) reflections of the cubic ZnTe phase, according to the JCPDS database, card no. 01-0582. Therefore, ZnTe thicker films are polycrystalline with the respective zinc-blende structures. It can be observed that features are amorphous bands at 5 and 10 min, and evolve to diffraction peaks at 15 and 20 min. We should note that, at these stages, the chemical reaction of Zn with Te was complete, even if the crystal quality was poor—no Zn metallic phase or shift on the peak position can be observed. The evolution of the crystallinity at different stages of growth suggests the existence of a relationship between the crystal quality and the layer thickness, as a result of different deposition times. An increase of the crystallite size with the thickness for ZnTe films was also reported by Aboraia et al. [23], where films of different thicknesses were obtained by plasma immersion O^−^ ion implantation. The Scherrer equation was used so we could get a quantitative idea about the crystal quality. This equation relates the peak broadening by the crystal quality in the following way [24]:

(1) $$\tau =\frac{k\lambda }{\beta cos\theta }$$

where k is the shape factor taken as 0.9, taking into account the spherical form of the grains, as shown in AFM images, λ = 0.154 nm is the wavelength of the X-rays and θ is the angular position. In the case of deposition times of 5 and 10 min, the diffraction features are amorphous and the Scherrer equation becomes inapplicable, at higher times (e.g., 15 and 20 min), the peak broadening on ZnTe (111) is 1.63° and 0.89°, respectively. Applying Equation (1), the mean crystallite size is 5.0 and 9.1 nm. At the same time, the position of the diffraction peaks remains unchanged, which indicates that the interplanar distances are preserved (e.g., d_111_ = 0.35 nm, d_220_ = 0.21 nm, and d_311_ = 0.18 nm according to Bragg’s law) for different deposition times. By applying the standard relation between the interplanar distances and the lattice constant for cubic crystals [25], it was found that the unit cell parameter is 0.61 nm. As a conclusion, the XRD findings indicate that the different deposition times lead to different sizes for the crystalline domains for ZnTe, while the unit cell parameter remains unchanged. This can be further ascribed to a constant lattice strain at different stages of formation for ZnTe films.

### 3.3. AFM Characterization

**Figure 3:**
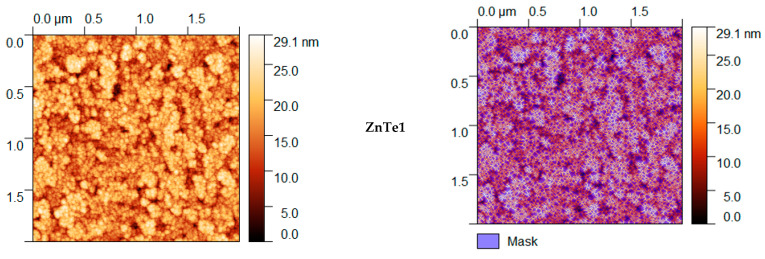

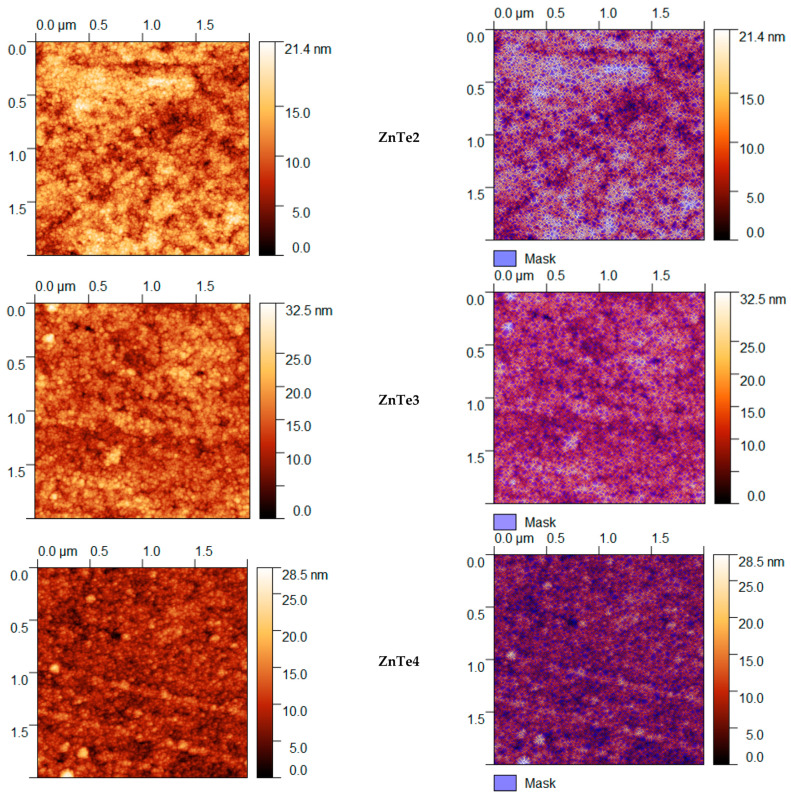
AFM characterization of ZnTe thin films with different thicknesses.

AFM characterization of ZnTe thin films with different thicknesses show that all films have a granular structure that evolve as thickness increases. As can be observed from the two-dimensional (2D) 2 μm × 2 μm images presented in Figure 3, the thickness increase led to surfaces with a much smaller z-range fact, meaning surface flattening and smoothening. Using Gwyddion software, manual surface segmentation was performed to evaluate medium grain parameter sizes (http://gwyddion.net/download/user-guide/gwyddion-user-guide-en.pdf, accessed on 30 August 2021). Specific surface segmentations for each kind of ZnTe film surface are presented in Figure 3. In the figure, the violet regions represent water shade masking of deeper film regions where the grain segmentation could not be clearly performed. Grain borders assigned by segmentation are shown in a red–brownish color. The segmentation was performed to identify the largest number of similar-sized grains present on the 2 μm × 2 μm surface scan. 

**Figure 4:**
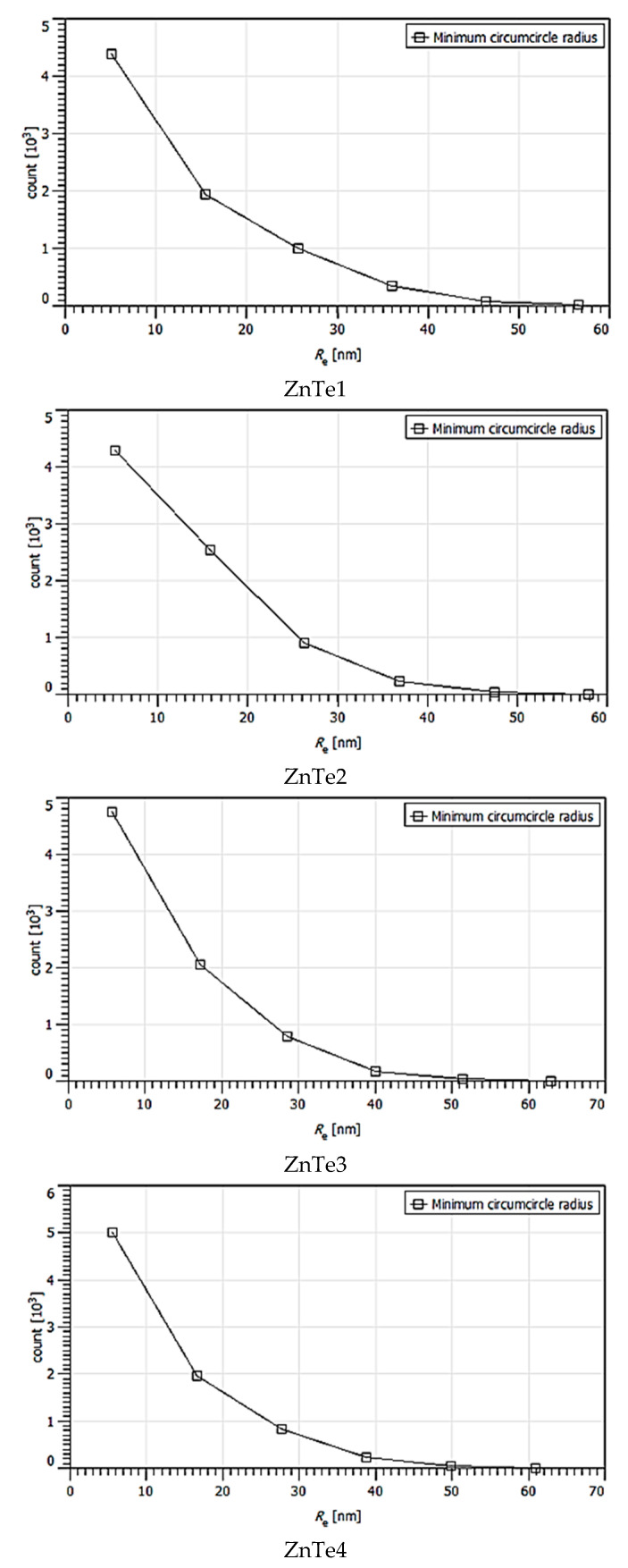
Minimum circumcircle radius value statistic variations onto surfaces of ZnTe1,2,3,4 thin films, presented in AFM images.

**Figure 5:**
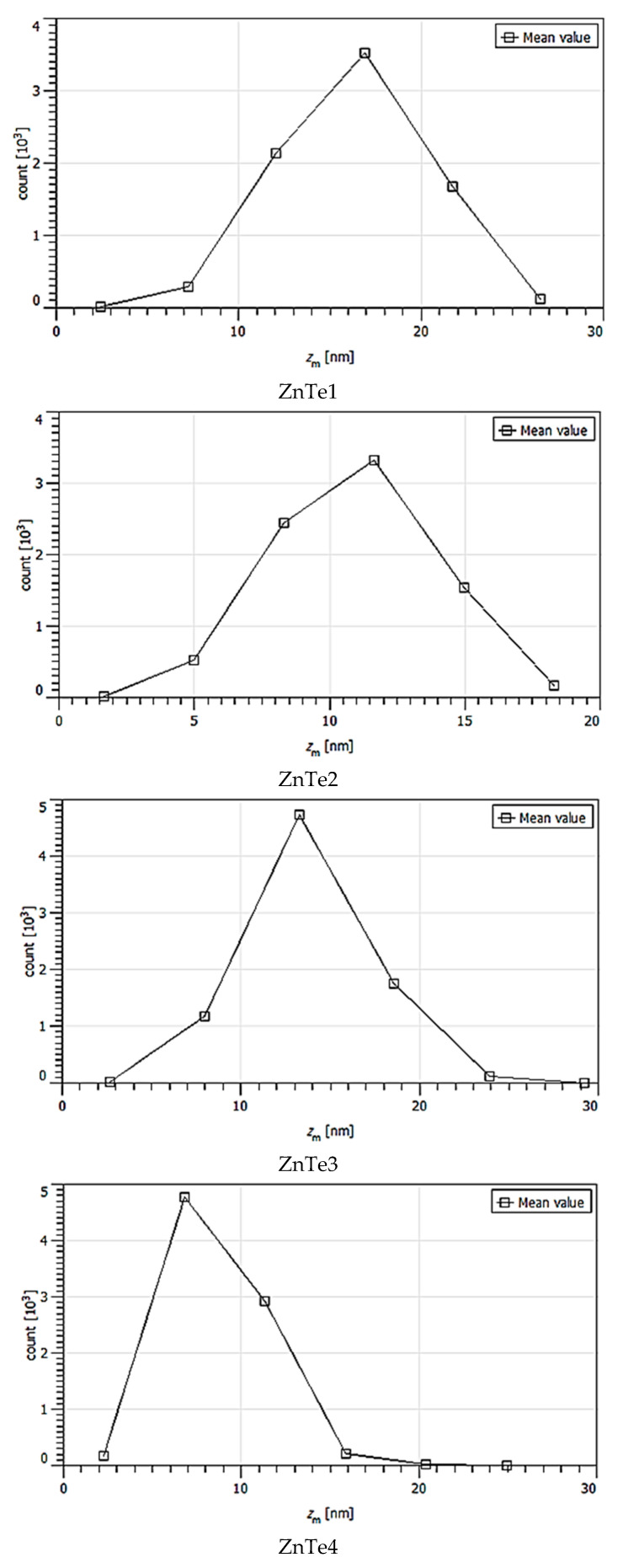
Mean value statistic variation onto surfaces of ZnTe1,2,3,4 thin films.

**Figure 6:**
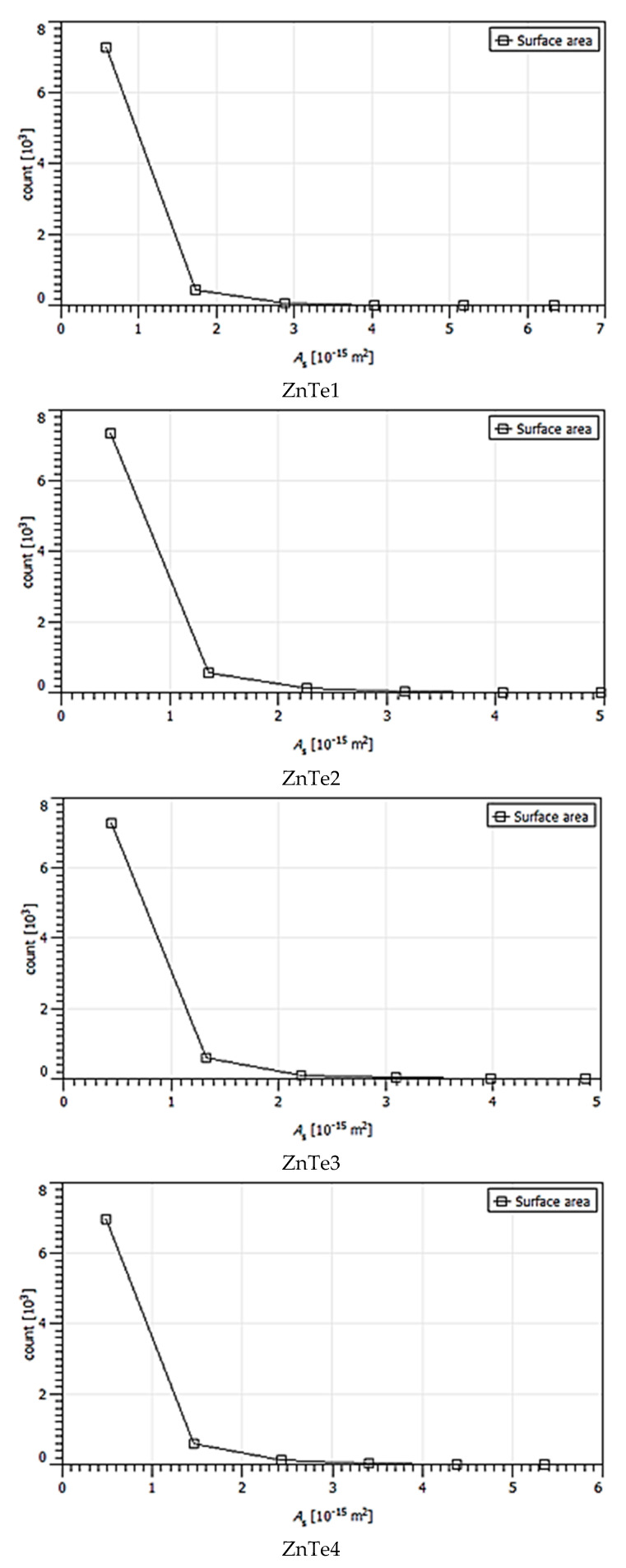
Surface area statistic variation of ZnTe1,2,3,4 thin films.

**Figure 7:**
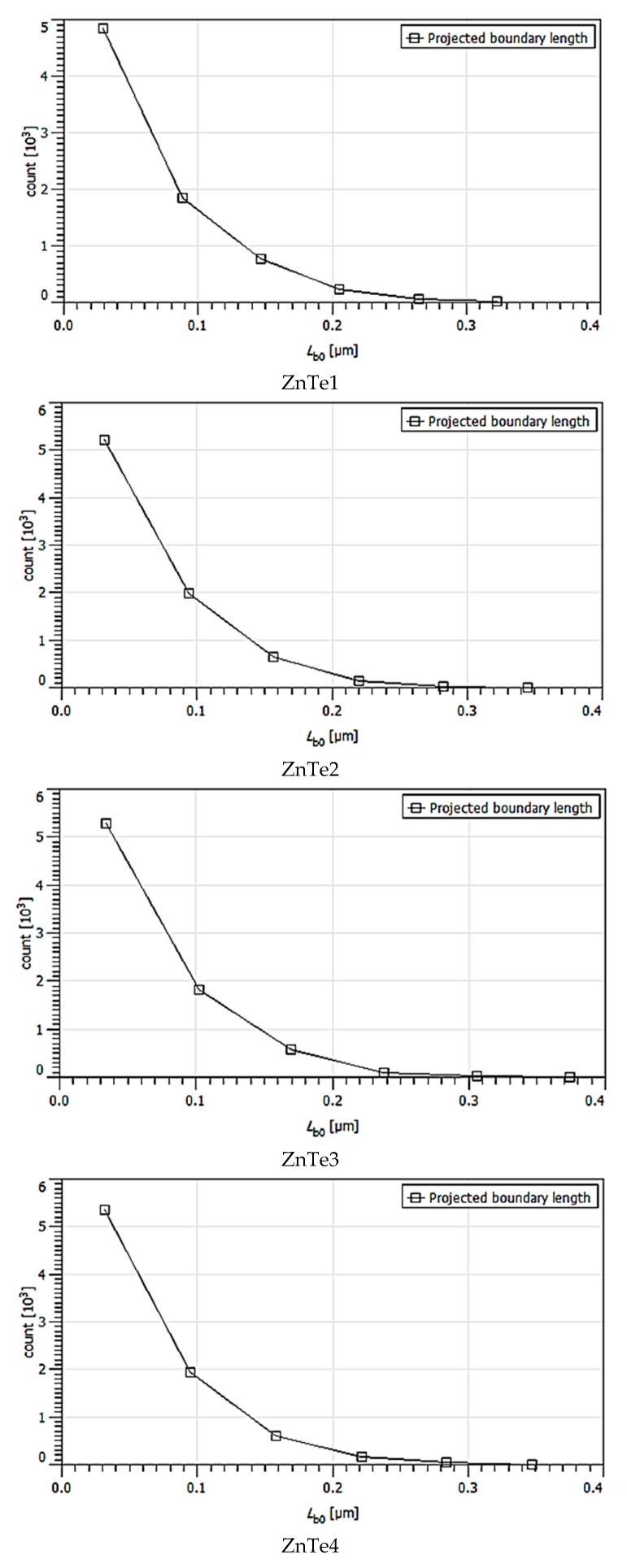
Projected boundary length statistic variation of ZnTe1,2,3,4 thin films.

**Figure 8:**
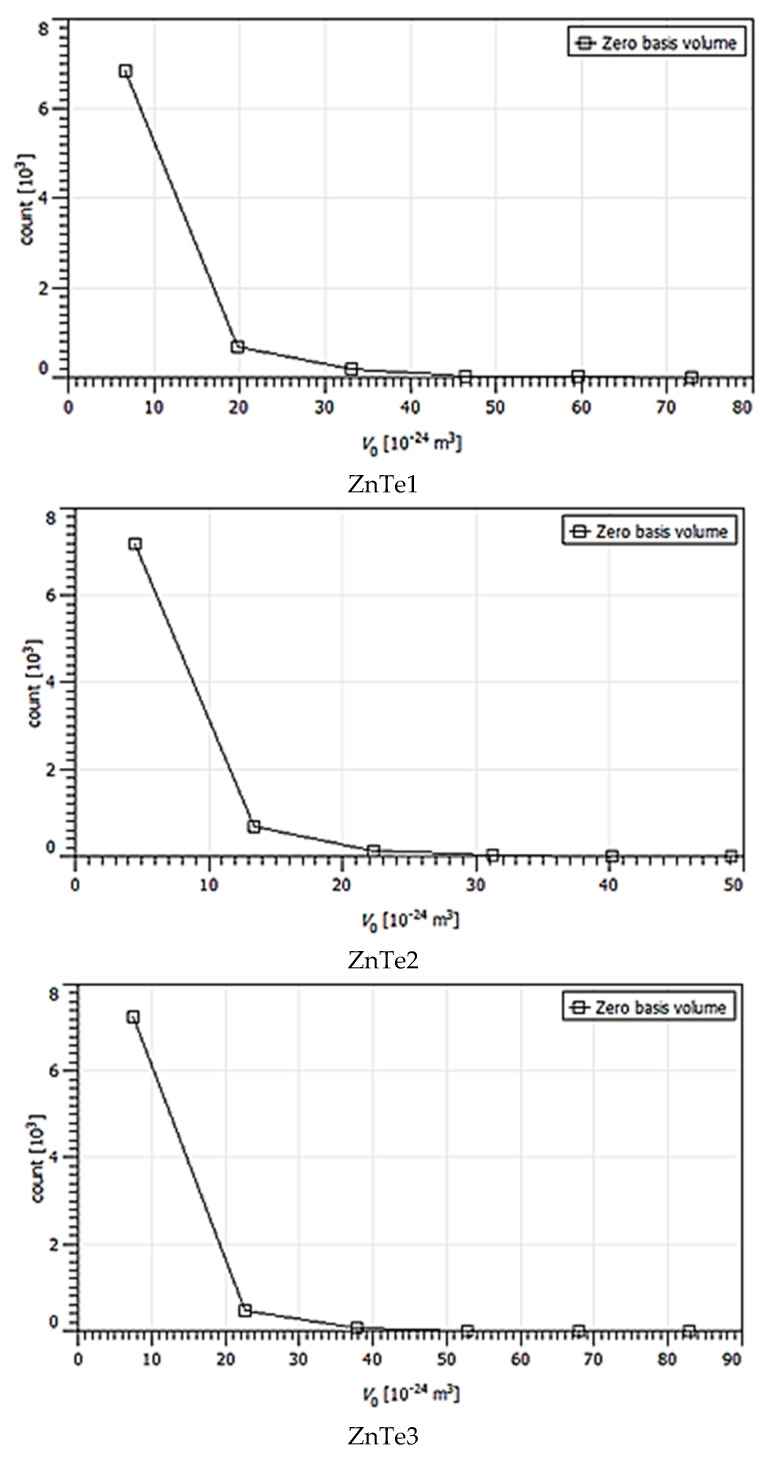

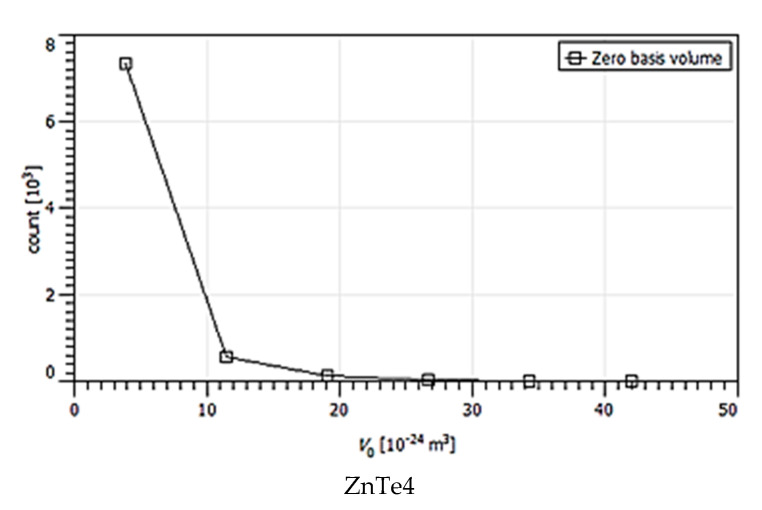
Zero basis volume statistic variation of ZnTe1,2,3,4 thin films.

The calculated majoritarian specific parameter size grains are presented in Table 1. These were chosen based on statistical distributions presented in Figure 4, Figure 5, Figure 6, Figure 7 and Figure 8. The calculus algorithms are open source, available at http://gwyddion.net/download/user-guide/gwyddion-user-guide-en.pdf, accessed on 30 August 2021.

Although the surface becomes flatter when the film thickens, it can be observed that the majoritarian grain on the surfaces remained the same, i.e., ~9–10 nm radius size for all of the films. The projected grain boundary values are also quite close—from 29 to 34 nm, while surface area and grain volumes are obviously more different due to strong surface texturing and a z-range drastic decrease. The surface-to-volume ratio is the amount of the surface area per unit volume of an object or collection of objects; in this case, the grains forming the film surface. It defines the relationship between the structures and functions in processes occurring through the surface and the volume of the film/layer. For the analyzed ZnTe thin films, the estimated surface to volume ratio was highest for the thinner film and was the lowest for the thicker. Additionally, the minimum circumcircle radius for each kind of grain detected on the surfaces, and the mean z-value were estimated for each above-presented AFM image, and are presented in the Figure 4, Figure 5, Figure 6, Figure 7 and Figure 8. Table 1 presents the mean calculated values for the ZnTe films surface characteristic grains (specific surface area, grain volume, grain boundaries lengths, and surface to volume ratios) that can be further connected with the film growth mechanisms and physical properties. Based on Figure 4, Figure 5, Figure 6, Figure 7 and Figure 8 that present the dimensional distribution of these surface characteristic parameter values for each of the films, the proper estimation of majoritarian grains is confirmed, and information regarding the other non-majoritarian grains (number and size ranges) present on the samples surfaces is provided. This offers the most complete kind of characterization regarding surface properties of the ZnTe films that one could obtain.

The Figure 4, Figure 5, Figure 6, Figure 7 and Figure 8 show the dimensional distributions of each parameter value onto the 2 μm × 2 μm surface scans (Figure 4).

**Figure 9:**
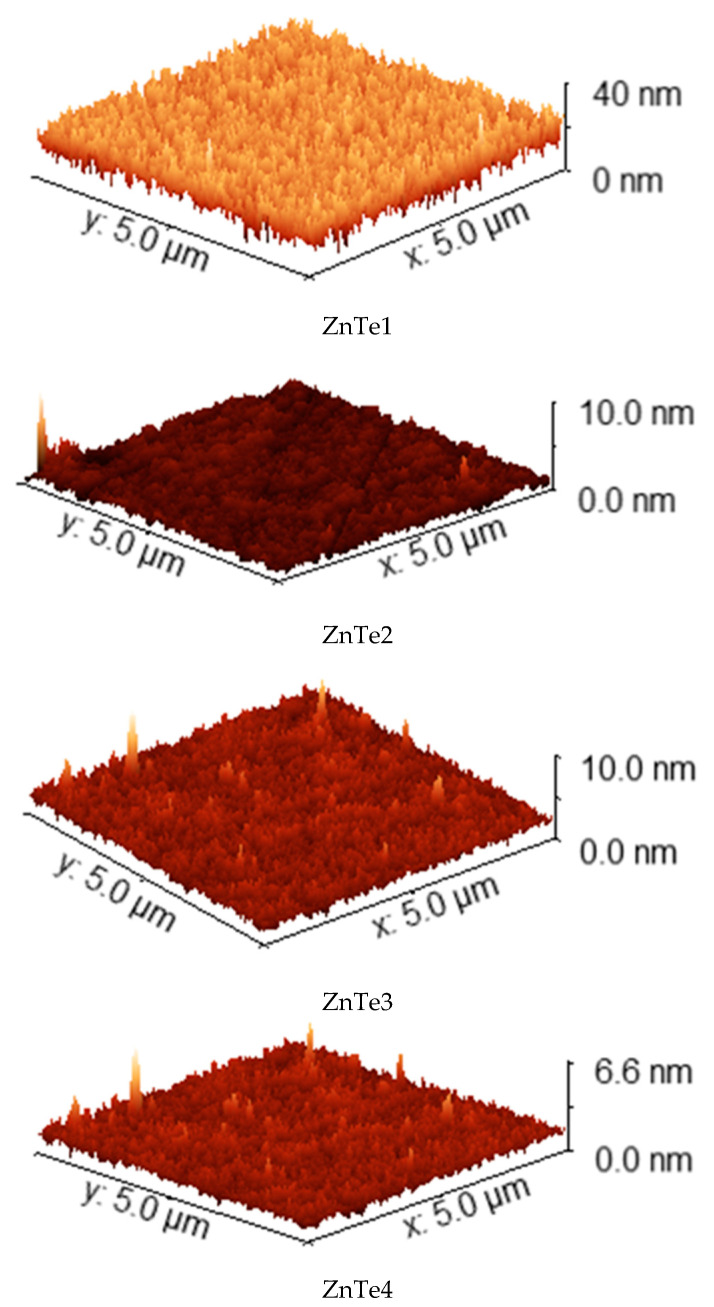
Examples of three-dimensional (3D) AFM images of ZnTe films.

To better analyze the surface roughness evolution, examples of three-dimensional (3D) AFM images of ZnTe films obtained from larger scan sizes (5 μm × 5 μm) are shown in Figure 9. The z-range of the specific 3D representations was chosen to be equal to the highest feature onto the surface, as a better way to observe the regularities and irregularities of the specific surface roughening process determined by film thickening.

It could be observed that the thicker films exhibit a waviness surface texture while the thinnest show pure grain distribution onto the surface. The growth time effect, onto surface structuring and morphology, is very strong. The thicker ZnTe film surfaces present valley regions, which become relatively smoother as the thickness increases. The cliff regions consist of spike structures that exhibit some orientation. The roughness parameters on surface morphology were estimated by analyzing the 5 μm× 5 μm topography images of the sample surface (Figure 9): average values, peaks, and valleys in the height direction Ra, average amplitude in the height direction Rq, and average characteristics in the height direction Rsk.

The average roughness Ra involve peaks and valleys, the mean height measured in the entire area, useful for detecting the profile height sample characteristics. Ra variation typically signifies a change in the growth process. It can be observed that Ra decreases as the film thickness increases. Root mean square (RMS) roughness Rq is the square root of the standard distribution in the surface profile from the average height. Sq is more sensitive than the average roughness Sa for large deviations from the mean plan and is the most commonly reported measurement of surface roughness. ZnTe thin film thickness increase leads to an Rq decrease with ~25%. Ten-point mean height roughness (Rz) is a height parameter, the difference between the average of the five highest peaks and five lowest valleys in the sample surface Rz [26]. Due to the change in surface morphology from uniform granular distribution to a combination of isolated tall grains and tight packaging of grains at increasing thickness, one can see that Rz varies randomly in our case. Rsk values represent the degree of bias of the roughness shape (asperity). It can be observed that the Rq and Ra decrease severely with a film thickness increase, leading to precise determination of skewness Rsk and ten-point mean height roughness Rz, as can be seen in the high deviations in Table 2. The decrease of all surface morphology parameters indicates an inhomogeneity decrease. As the thickness increases, the average roughness Ra and RMS roughness Rq surface roughness decreases, restricting charged species to be adsorbed on the polycrystalline film. These changes may be related to the rf magnetron sputtering deposition process, which can stimulate the migration of grain boundaries and create more grains during the growth process. Moreover, at a high deposition rate, the supplementary energy encourages the atoms to acquire and occupy the correct site in the crystal lattice, such that the grains with lower surface energy will grow. These correlate well with the XRD observations and the fact that thinner films are amorphous while the thicker become crystalline. Further studies on intermediate growth times will be performed for a better solving of thin film growth mechanism evolution.

### 3.4. Optical Characterization

#### 3.4.1. Ellipsometry

**Figure 10:**
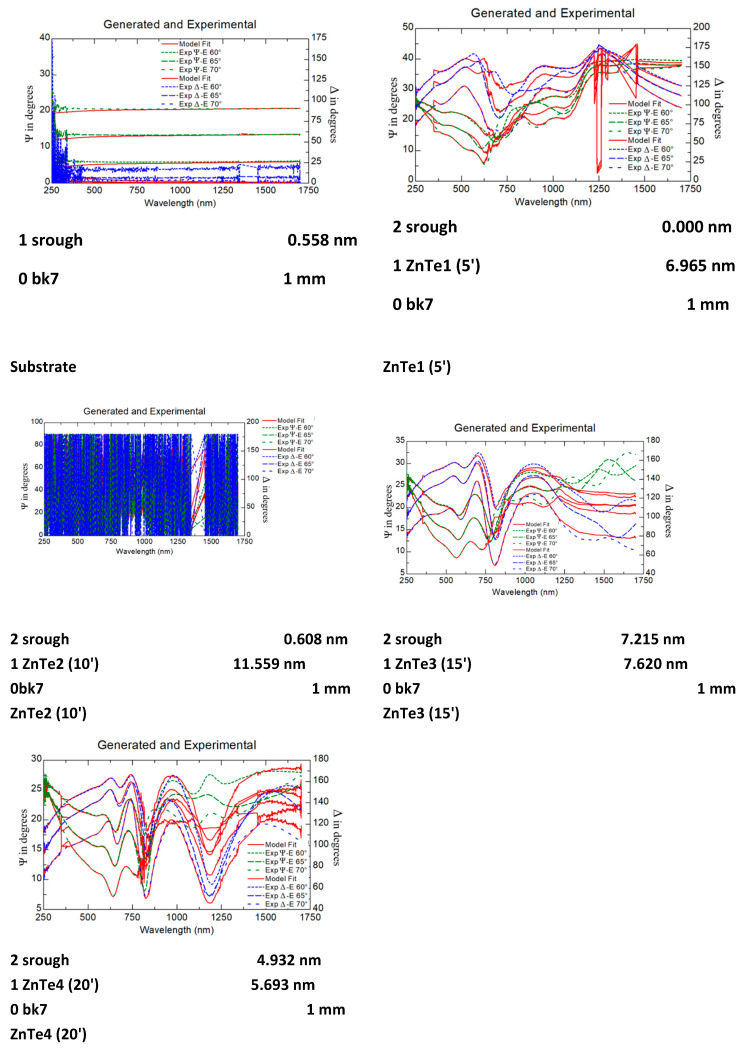
Theoretical and experimental ellipsometry data (Δ and Ψ) of ZnTe thin films at three incidence angles.

Ellipsometry measurements were performed onto all ZnTe samples. Using the WVASE 32 software package, the simulation of the theoretical curves was performed. The obtained parameters were Ψ and Δ in the spectral range 250–1700 nm, scanning with a step of 2 nm. The analysis of the samples was performed at three incidence angles (60°, 65°, and 70°) with a step of 5° as described in references [27,28,29,30]. A ZnTe semiconductor model was selected according to the reference [30] for better measurement of bandwidth absorption, which is very important for the design of solar cells [29]. These aspects demonstrate the complexity of the thin film structures and influence of n, k parameters for many A2-B6 compounds [31,32,33,34,35]. As for the SE system, it was equipped with software for control and simulation of theoretical curves using a theoretical model [28]. The optical model was elaborated based on three layers: glass substrate, the ZnTe layer simulated using the Zinc telluride mathematical model in the WVASE database,, adjusting the thickness to fit the data, and the third layer—“srough”. The zinc telluride mathematical model in the V VASE database is a GenOsc Tauc-Lorentz model. The model is fitted to minimize the mean square error (MSE) and reach a normal fit. Normal fit is reached by iterative approximations. The number of required iterations differs for each of the samples. Details regarding the simulation steps and models can be found in [27]. Film thickness and film roughness vary due to growth conditions. The scanning of the samples (ZnTe) and the substrate (BK7 Glass) were performed followed by the simulation of the theoretical curves with the help of the software, as can be seen from the examples presented in Figure 10.

Figure 10 presents the SE measurements and theoretical modeling at three incidence angles (60°, 65°, and 70°) in the spectral range 250–1750 nm for estimation of optical roughness of the substrate and the ZnTe films analyzed during this study. Good qualitative agreement was found between the SE measurements and the theoretical simulations. The fitted SE spectra deviate significantly from the measured ones for the thinner films. These differences may be associated with the deviations of film stoichiometry and crystallinity in the various stages of growth from the ideal models. The differences are smaller for the thicker films. Further studies for better correlations are ongoing.

Table 3 represents a comparative presentation of the roughness measured by SE and RMS measured by AFM. The observed difference is generated by the different measurement principles of SE and AFM. SE measures the effect of roughness at the atomic size. The value of the RMS roughness is influenced from the peak to the height of the valley being approximately twice as high as the one estimated by SE measurements.

#### 3.4.2. Optical Spectroscopy and Bandgap

**Figure 11:**
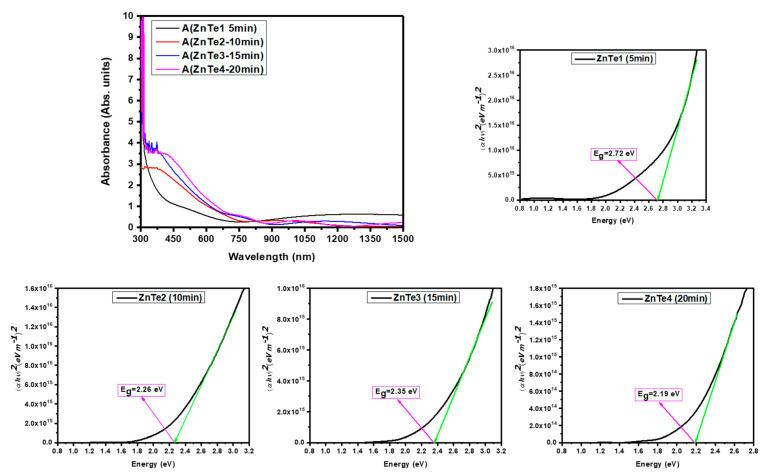
Examples of UV-Vis absorption spectra and of optical bandgap energy estimation using Tauc Plots.

To study the optical properties of the fabricated samples, UV-Vis absorption measurements were performed. The UV-Vis absorption spectra of each sample, as well as the extrapolation used to calculate their optical bandgap using Tauc plots were obtained. Since ZnTe is a direct bandgap semiconductor, (αhν)^2^ was used to calculate the bandgaps. Representative examples of some absorption spectra and Tauc plots derived from the ZnTe thin film analysis are presented in Figure 11.

Bandgap energy (Eg) values obtained for the optimized materials are presented in Table 4.

**Figure 12:**
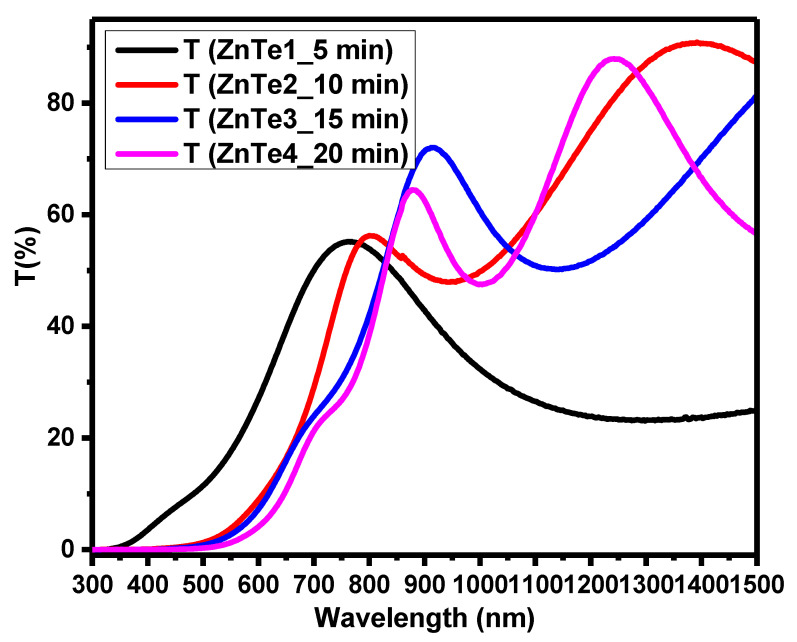
UV-Vis transmission spectroscopy spectra of ZnTe thin films with different thicknesses.

It was observed that the increased thickness leads to a decrease of optical transmittance in the visible region and the presence of the fringes in the NIR region of the electromagnetic spectrum (see Figure 12). The calculated bandgap values slightly decrease with the thickness increase.

## 4. Conclusions and Perspectives

ZnTe thin films with different thicknesses on BK7 glass substrates were grown by the rf magnetron sputtering technique, using time as a variable growth parameter. All other deposition process parameters were kept constant. The fabricated thin films with mean thickness, ranging from 75 to 461 nm, were characterized using electron microscopy, X-rays diffraction, atomic force microscopy, ellipsometry, and UV-Vis spectroscopy, to evaluate their structures, surface morphology, topology, and optical properties. By using SEM to measure the obtained film thickness, it was found that the deposition time increase leads to a larger growth rate. This determines significant changes on ZnTe thin film structuring and surface morphology. Characteristic surface metrology parameter values vary, and surface texturing evolves with thickness increase, correlating well with XRD analysis findings that thinner films are amorphous, and at least 10 min of growth is needed for the crystalline material to start to form. Larger thickness films show diffraction peaks located at 25.24°, 42.34°, and 49.59°, which corresponds unambiguously to (111), (220), and (311) reflections of the cubic ZnTe phase, according to the JCPDS database with card no. 01-0582. For these films, the mean crystallite sizes are ~5 and ~9 nm, smaller than the surface grain size, estimated from the AFM analysis to be ~9 nm radius, i.e., ~18 nm diameter. The crystallite size shows values comparable to the ZnTe exciton radius estimated at ~6–7 nm [36], fact that opens interesting supplementary research perspective on size quantum effects on optical properties of such ZnTe films grown onto various substrates. Optical bandgap energy values slightly decrease as the thickness increases, while the mean grain radius remains almost constant at ~9 nm and the surface to volume ratio per grain decreases by two orders of magnitude. To our knowledge, this study is the first attempt that thoroughly considers the correlation of film thickness with ZnTe structuring and surface morphology characteristic parameters. It adds value to the existing knowledge on ZnTe thin film fabrication and their physical properties, tailored for various applications, including photovoltaics. There are ongoing studies regarding the correlation between structure and surface morphology, with the observed optical properties; this will lead to future scientific reports.

## Figures and Tables

**Table 1:** The size and specific parameters for the grains.

Sample	Mean Surface Area m^2^	Number of Grains Used for the Surface Area Estimation	Mean Volume m^3^	Number of Grains Used for Volume Estimation	Grain Boundaries nm	Number of Grains Used for Grain Boundaries Estimation	Mean Grain Radius nm	Number of Grains Used for Grain Radius Estimation	Surface Area/ Volume m^−1^
**ZnTe1**	4.87E−16	6979	3.61E−26	7163	29.37	4851	9.46	5772	1.35E + 10
**ZnTe2**	4.42E−16	7270	2.26E−24	5773	31.36	5208	9.39	5884	1.95E + 8
**ZnTe3**	5.76E−16	7272	2.16E−25	7442	33.97	5283	9.95	6033	2.67E + 9
**ZnTe4**	4.52E−16	7337	2.39E−24	5432	31.59	5357	9.68	6210	1.89E + 8

**Table 2:** Calculated roughness parameters on surface morphology.

	ZnTe1 (nm)	ZnTe 2 (nm)	ZnTe 3 (nm)	ZnTe 4 (nm)
**Rq**	4.08	3.45	3.29	3.09
**Grain-wise Rpv**	39.54	24.07	60.49	37.95
**Ra**	3.30	2.77	2.46	2.32
**Rsk**	0.18	0.04	−1.33	−0.92
**Rz**	36.32	23.52	54.33	36.03
**Rku**	2.87	2.79	14.55	6.13

**Table 3:** Surface roughness measured by SE and AFM on ZnTe samples that have different deposition times.

Sample	Type of Substrate	Deposition Time (min)	Roughness Measured by SE (nm)	RMS Roughness AFM (nm)		Thickness Nonuniformity (%)	MSE
				2 × 2 µm ^2^	5 × 5 µm ^2^		
**ZnTe1 (5’)**	BK7	5	0.000	4.2	4.1	38.851%	15.01
**ZnTe2 (10’)**	BK7	10	0.608	3.1	3.5	63.064%	0.2747
**ZnTe3 (15’)**	BK7	15	7.215	3.4	3.3	100%	7.693
**ZnTe4 (20’)**	BK7	20	4.982	2.5	3	100%	19.55

**Table 4:** Bandgap values.

Sample	Bandgap Eg(eV)
**ZnTe1**	2.72
**ZnTe2**	2.26
**ZnTe3**	2.35
**ZnTe4**	2.19

## Data Availability

The raw and processed data required to reproduce these findings cannot be shared at this time due to technical or time limitations. The raw and processed data will be provided upon reasonable request to anyone interested anytime, until the technical problems will be solved.
